# Intermittent sprint performance in the heat is not altered by augmenting thermal perception via L-menthol or capsaicin mouth rinses

**DOI:** 10.1007/s00421-018-4055-0

**Published:** 2018-12-22

**Authors:** O. R. Gibson, J. G. Wrightson, M. Hayes

**Affiliations:** 1 Centre for Human Performance, Exercise and Rehabilitation, College of Health and Life Sciences, Brunel University London, Uxbridge, UK; 2 Division of Sport, Health and Exercise Sciences, Department of Life Sciences, College of Health and Life Sciences, Brunel University London, Uxbridge, UK; 3 Faculty of Kinesiology, University of Calgary, Calgary, Canada; 4 Environmental Extremes Laboratory, University of Brighton, Eastbourne, UK

**Keywords:** Cooling, Heat stress, Sprint performance, Temperature, Thermal comfort, Thermal sensation, Thermal perception

## Abstract

**Purpose:**

Cooling sensations elicited by mouth rinsing with L-menthol have been reported as ergogenic. Presently, responses to L-menthol mouth rinsing during intermittent sprint performance (ISP) in the heat are unknown and the impact of increased thermal perception on ISP via capsaicin has also not been quantified. This experiment aimed to identify whether eliciting cooling/warming sensations via L-menthol/capsaicin would alter ISP in the heat.

**Method:**

Fourteen participants (mass = 72 ± 9 kg, $\dot {V}{{\text{O}}_{2{\text{peak}}}}$ = 3.30 ± 0.90 L min^−1^), undertook four experimental trials, involving 40 min of ISP in hot conditions (40.2 ± 0.6 °C, 42 ± 2% R.H.) with mouth rinsing (25 mL, 6 s) at the protocol onset, and every 10 min thereafter. Cooling (0.01% L-menthol; MEN), warming (0.2% capsaicin; CAP), placebo (0.3 sham-CHO; PLA), and control (water; CON) mouth rinses were utilized. Performance was quantified via power (PP) and work done (WD) during sprints. Heart rate (HR), core (*T*_rec_) and skin (*T*_skin_) temperature, perceived exertion (RPE), thermal sensation (*T*_sens_), and comfort (*T*_com_) were measured at 10 min intervals. Sweat rate (whole-body sweat rate) was calculated from ∆mass.

**Result:**

PP reduced over time (*P* < 0.05); however, no change was observed between trials for PP or WD (*P* > 0.05). *T*_com_ increased over time and was lower in MEN (2.7 ± 1.1; *P* < 0.05) with no difference between CAP (3.1 ± 1.2), PLA (3.2 ± 1.3) and CON (3.1 ± 1.3). RPE, *T*_sens_ HR, *T*_rec_, and *T*_skin_ increased over time (*P* < 0.05) with no between trial differences (*P* > 0.05).

**Conclusion:**

Despite improved thermal comfort via L-menthol, ISP did not improve. Capsaicin did not alter thermal perception or ISP. The reduction in ISP over time in hot conditions is not influenced by thermal perception.

**Electronic supplementary material:**

The online version of this article (10.1007/s00421-018-4055-0) contains supplementary material, which is available to authorized users.

## Introduction

In comparison to equivalent exercise demands in temperate conditions, heat stress impairs endurance (Kenefick et al. 2007) and intermittent (Girard et al. 2015) exercise performance. In cycling (Racinais et al. 2015b) and running (Guy et al. 2014) modalities, continuous intensity/aerobic endurance-type exercise performance diminishes with increased environmental temperature along a continuum once the magnitude of heat stress increases above preferable ambient temperatures of 10–15 °C (Galloway and Maughan 1997). Impaired performance of intermittent sprint activity replicating team sports, e.g., football/soccer, rugby league and union, field hockey, basketball and netball, during heat stress has been characterized (Hayes et al. 2014), albeit to a lesser extent than continuous exercise in spite of intermittent/repeated sprint exercise providing a greater heat strain (Maxwell et al. 1996). It has been reported that football/soccer players performing at the 2014 FIFA World Cup demonstrated a reduction in the number of sprints and total distance covered under high heat stress vs moderate or low heat stress (Nassis et al. 2015), an observation shared by others (Konefal et al. 2014; Watanabe et al. 2017). Conversely, within that same study, it was identified that technical parameters (rate of successful passes) were equal or enhanced in high heat stress vs moderate or low stress matches (Nassis et al. 2015), suggesting that ‘pacing’ of high-intensity work is occurring either in response to physiological or perceptual stimuli (Girard et al. 2017).

Detrimental performance under heat stress is closely related to the elevations in cardiovascular strain under heat stress (González-Alonso et al. 2008), which occur to attenuate elevations in core/deep body temperature via elevated skin blood flow and redistribution of cardiac output (González-Alonso 2012), ultimately to maximize evaporative cooling (Candas et al. 1979). Two experimental games of football played in hot (~ 43 °C) or temperate (~ 21 °C) conditions evidenced these responses with muscle and core temperature ~ 1 °C higher in the hot games (Mohr et al. 2012; Nybo et al. 2013). These temperature responses were not accompanied by elevated cardiovascular strain. However, this is likely a response to aforementioned reductions in physical performance whereby distance covered (− 7%) and high-intensity running (− 26%) were lower in the hot conditions, presumably to moderate the magnitude of cardiovascular strain, i.e., the players were incurring similar physiological strain for reduced work. In a follow-up study utilising the same data (Nybo et al. 2013), no difference in maximal voluntary contraction, voluntary activation, and peak twitch torque, nor the magnitude of glycogen depletion occurred in the football matches played in the hot or temperate conditions, suggesting that these pathways are not directly contributing to reduced physical performance in the heat. Thermally driven pacing during intermittent team sport type activity has been evidenced in the laboratory, whereby relative to temperate conditions, hot-wet and hot-dry conditions elicit an earlier and greater reduction in peak power output during 40 min of high-intensity sprints (Hayes et al. 2014). The conscious/subconscious pacing of performance becomes apparent given equality of performance during the final sprint/end spurt across conditions inspite of earlier reductions in performance (Hayes et al. 2014). It has previously been identified that cooling at a physiological and perceptual level (Castle et al. 2006; Duffield et al. 2010) can enhance self-paced exercise performance under heat stress in laboratory conditions. This suggests, in these scenarios, that attenuated physiological or perceived temperature is ergogenic and, therefore, has the potential to improve intermittent sprint performance. Conversely, under conditions of equivalent core temperature, deception of temperature (Castle et al. 2012) and subsequent reductions in RPE leads to an improvement in performance, suggesting that perception of temperature is at least in part responsible for performance detriments rather than the physiological temperature alone (within the range of internal temperatures elicited by these experiments).

Eliciting alterations in thermal perception in the absence of any physiological differences in temperature has been termed ‘non-thermal cooling’ or ‘non-thermal warming’. Non-thermal cooling can be administered by topical, facial applications of L-menthol [an activator of the transient receptor potential (TRP) ion-channel Melastatin 8 (TRPM8) (Montell and Caterina 2007)] to elicit alterations in thermal sensation to that of fan cooling, and capsaicin [an activator of the TRP ion-channel Vanilloid 1 (TRPV1) (Montell and Caterina 2007)] has been used to elicit non-thermal warming eliciting equivalent changes in thermal perception to that of a heater (Schlader et al. 2011). Non-thermal cooling sensation of L-menthol improved mean power output to the same extent as the actual cooling (+ 21% during RPE-clamped exercise), whilst capsaicin and actual warming reduced power output relative to cooling trials (Schlader et al. 2011). These data inform that self-paced fixed-intensity exercise performance such as time trials or time to task failure/exhaustion in the heat can be modulated by thermoregulatory behavior. Given the difficulties in applying non-thermal cooling via facial applications during fixed-intensity exercise performance, the ergogenic potential of non-thermal cooling via L-menthol in the form of a mouth rinse has received a significant attention. A recent review concluded that a mouth rinse or a beverage containing L-menthol during endurance exercise in the heat is beneficial for performance (Stevens and Best 2017). It was stated that ergogenic benefits for performance are contingent on altering thermal perception/perceived exertion (Mündel and Jones 2010; Stevens et al. 2016, 2017; Flood et al. 2017; Jeffries et al. 2018). This notion is supported by the observation that protocols which did not report altered perceptual responses following oral L-menthol do not demonstrate a performance enhancement (Sönmez et al. 2010; Riera et al. 2014, 2016). At present, only one experiment has investigated the use of L-menthol [combined with cool fluid (0.2 or 3.0 °C)] during interval-type activity (five repetitions of 4 km cycle and 1 km running TT performance), whereby no ergogenic improvement was attributable to L-menthol (Trong et al. 2015). To the authors’ knowledge, no data exist investigating the use of L-menthol during intermittent sprint activity replicating that of team sports. This is surprising given that the natural breaks in play during team sport performance would facilitate mouth rinsing at the same time as habitual drinking (Garth and Burke 2013), something not as plausible in a continuous, fixed-intensity competitive endurance event such as track running/cycling.

The aim of this experiment was to determine whether eliciting a cooling sensation via oral L-menthol or a warming sensation via oral capsaicin would alter intermittent sprint performance in the heat in comparison to control and placebo oral solutions. It was hypothesized that L-menthol would reduce (enhance) thermal sensation and improve performance, whilst capsaicin would increase (diminish) thermal sensation and decrease performance.

## Methods

### Participants

Fourteen healthy, non-heat-acclimated, trained team sports players volunteered to participate in the study (Participant characteristics are presented in Table 1). Initially, 16 participants volunteered and commenced the experiment; however, two withdrew having completed two and three visits for reasons unrelated to the experiment. Confounding variables of caffeine and alcohol consumption 24 h prior to testing and prolonged thermal, e.g., exercise-heat acclimation protocols, repeated sauna or hot tub use, or hypoxic exposures, e.g., altitude training in the 6 weeks prior to testing, were all controlled for in line with the previous work involving exercise-heat stress and intermittent sprinting (Hayes et al. 2014; Gibson et al. 2014). Following institutional ethics approval (2732-MHR-Jul/2016-3430-2) and full description of experimental procedures, all participants completed medical questionnaires and provided written informed consent following the principles outlined by the declaration of Helsinki of 1975, as revised in 2013.

**Table 1:** Mean ± SD participant characteristics (*n* = 14; 11 males; 3 females)

Variable	Mean ± SD
Age (years)	24 ± 3
Height (cm)	175 ± 12
Mass (kg)	71.6 ± 8.8
Body surface area (m^2^)	1.86 ± 0.17
Body mass index (kg m^2^)	23.4 ± 2.2
Body fat (%)	11.6 ± 3.1
Maximal oxygen uptake (L min^−1^)	3.29 ± 0.89
Maximal oxygen uptake (mL kg^−1^ min^−1^)	46.2 ± 12.9
Power at maximal oxygen uptake (W kg^−1^)	3.8 ± 1.3
Power eliciting 35% $\dot {V}{{\text{O}}_{2{\text{peak}}}}$ (W kg^−1^)	1.3 ± 0.5

### Experimental design

The protocol consisted of five visits. The first visit was an incremental test to determine peak oxygen uptake ($\dot {V}{{\text{O}}_{2{\text{peak}}}}$) and maximum aerobic power output (*W*_max_). During the same visit, the subjects completed a familiarization trial of 20 min of a cycling intermittent sprint protocol [CISP; The full CISP is 40 min in duration, and involves 20 × 2 min blocks of 10 s passive rest, a 6 s sprint against 7.5% of body mass, and 104 s of active recovery at a power eliciting 35% $\dot {V}{{\text{O}}_{2{\text{peak}}}}$ determined from regression of $\dot {V}{{\text{O}}_2}$ against power from the incremental test (Castle et al. 2011)] in hot conditions [40 °C, 50% relative humidity (RH)]. This served to minimize the negative effects of initial heat exposure and any subject learning effect associated with the protocol (Hayes et al. 2014). Then, a minimum of 48 h later, in a randomized and cross-over design, participants commenced the experimental trials (visits 2–5). Female participants performed experimental trials during the follicular phase of the menstrual cycle (Mee et al. 2015; Lei et al. 2016). Trial order was counterbalanced using a Latin square based on 16 participants. Each experimental visit to the laboratory was separated by a minimum of 48 h to allow for a full-recovery between trials, and to mitigate against physiological adaptation to the heat (Gill and Sleivert 2001). All exercise tests were carried out on a friction braked cycle ergometer (Monark 724, Vansbro, Sweden), operating in a pedal-rate independent mode.

Experimental trials involved participants performing a CISP in a heat chamber maintained at ~ 40 °C, ~ 50% RH. In the experimental trials, subjects periodically swilled an L-menthol, capsaicin, water, or orange-flavored placebo solution at four time points. Each mouth rinse was 25 mL in volume and administered at rest and at 10 min intervals thereafter, i.e., after every 5th sprint during the 10 s passive rest phase of the CISP protocol. The participants were instructed to swill/gargle the solution around the mouth for 5 s and then expectorate the solution into a bowl without swallowing. The menthol solution (MEN) was a 0.01% concentration of L-menthol crystal ground and dissolved in distilled water (≥ 99% food grade L-menthol, Sigma-Aldrich, UK) in line with the previous experiments (Mündel and Jones 2010; Stevens et al. 2016). The capsaicin solution (CAP) was administered at a concentration of 0.2% with the capsaicin containing red pepper sauce (Tabasco Habanero Sauce, McIlhenny Co., Avery Island, CA, USA) diluted in distilled water. These differences in concentrations were used based upon pilot data which identified them to elicit an equal magnitude of perceptual change in thermal sensation of the oral cavity + 0.5 (MEN) or − 0.5 (CAP) on the scale (Toner et al. 1986) at rest, in thermoneutral conditions. Orange-flavored fruit squash (Tesco Ltd, UK) solution mixed with distilled water to create a 0.5% (0.3 g/100 mL) placebo-CHO solution (PLA) (Carter et al. 2004). Distilled water served as the control (CON). To minimize the impact of drink temperature on perceptual or physiological responses (Lee and Shirreffs 2007), all fluids were maintained to the temperature within the heat chamber (~ 40 °C), this also served to ensure that the L-menthol crystals remained dissolved in water and ensured minimal pre/per-cooling effect given the close proximity of the rinse temperature to that of the oral cavity.

### Preliminary testing

Prior to the initial assessment of $\dot {V}{{\text{O}}_{2{\text{peak}}}}$ and *W*_max_ in the preliminary trial, standing height (cm) was measured via a stadiometer and nude-body mass (kg) was recorded following self-reported measurement in a private bathroom (SECA 875 scale, Birmingham, UK). These data were used to calculate body surface area (Du Bois and Du Bois 1916). Body density was also calculated using calipers (Harpenden, Burgess Hill, UK) and a four-site skinfold calculation (Durnin and Womersley 1974). This was later used to calculate body fat [%, (Siri 1956)].

The incremental test was conducted in temperate lab conditions (21 °C, 50% RH). Starting intensity was set at 80 W with resistance subsequently applied to the flywheel to elicit a 24 W min^−1^ increase at the constant cadence of 80 rpm. Expired metabolic gas was measured using online gas analysis (Oxycon Pro, Jaeger GmbH or Metalyser Sport, Cortex, Leipzig, Germany); the $\dot {V}{{\text{O}}_{2{\text{peak}}}}$ was considered as the highest $\dot {V}{{\text{O}}_2}$ obtained in any 10 s period. Heart rate (HR; b min^−1^) was recorded continually during all exercise tests by short-range telemetry (Polar Electro Oyo, Temple, Finland). Saddle position was adjusted by the participant to their preferred cycling position and remained unchanged for all experimental trials. Experimental workloads (i.e., active recovery at 35% of $\dot {V}{{\text{O}}_{2{\text{peak}}}}$) were subsequently calculated using linear regression utilising power: $\dot {V}{{\text{O}}_2}$ data collected following the incremental test.

After 25 ± 5 min recovery, a 20 min stabilization period (seated rest), and ten stages (20 min) of the CISP were completed in the heat chamber (~ 40 °C, ~ 50% RH). Following stabilisation, a standard warm-up [5 min at 95 W (80 rpm) and two 30 s bouts at 120 W (100 rpm) with 30 s rest in between (Hayes et al. 2012, 2014)] were followed by the 10 × 2 min blocks of the CISP, i.e., ten instances of 10 s passive rest, a 6 s sprint against 7.5% of body mass, and 104 s of active recovery at a power eliciting 35% $\dot {V}{{\text{O}}_{2{\text{peak}}}}$ (Castle et al. 2011).

### Experimental procedure

Each experimental trial was conducted at the same time of day (within participants) to control for the effects of circadian variation in performance (Drust et al. 2005). To minimize differences in starting muscle glycogen concentrations between visits, subjects recorded their diet in the 24-h period before the second visit and were instructed to follow the same diet before each subsequent visit. Energy or macronutrient intake was not quantified in the experiment. Participants consumed 500 mL of water 2 h before all preliminary and experimental exercise sessions to ensure adequate hydration. Once participants were deemed euhydrated [i.e., urine osmolality (Alago Vitech Scientific, Pocket PAL-OSMO, UK) was < 700 mOsm kg^−1^ H_2_O (Sawka et al. 2007)], they were subsequently able to commence further preliminary and experimental procedures. Following confirmation of adequate hydration, in private, participants measured their nude-body mass (NBM) inserted a single-use disposable rectal thermistor (Henleys Medical, UK, Meter logger Model 401, Yellow Springs Instruments, Yellow Springs, Missouri, USA) 10 cm past the anal sphincter to facilitate the measurement of rectal temperature (*T*_rec_). Skin temperature (*T*_sk_) was measured using a data logger (Squirrel Meter Logger, Grant Instruments, Cambridge, UK) and skin thermistors secured at four sites (pectoralis major muscle belly, lateral head of triceps brachii, rectus femoris muscle belly, and lateral head of the gastrocnemius) on the right-hand side of the body using 6 cm × 7 cm transparent Tegaderm patches (3M, UK). Weighted mean skin temperature was determined using a four-site formula (Ramanathan 1964). Heart rate was recorded continually in the same manner as the preliminary test.

Participants then mounted a cycle ergometer located inside the environmental chamber where conditions were consistent across trials (~ 40 °C, ~ 50% RH) and performed the warm-up followed by the full 40 min CISP. During the CISP, the Monark Anaerobic software (Monark Anaerobic Wingate Software, Version 1.0, Monark, Vansbro, Sweden) recorded peak power (PP), mean power (MP), and work done (WD) at a sampling frequency of 50 Hz, during each sprint within of each 2 min stage of the CISP. Peak power was recorded as the highest recorded power output value for each sprint; MP was determined as the average power output from all values recorded during the 6 s sprint. WD was calculated cumulatively over the whole protocol. Physiological measurements were recorded at rest (0 min) and then 60 s into the active recovery phase after every 5th sprint (every ~ 10 min) thereafter. Perceptual responses were also recorded at 10 min intervals with this frequency least likely to cause non-experimental artifacts (Corbett et al. 2009). Perceptual responses included whole-body thermal comfort (*T*_com_) and thermal sensation (*T*_sen_) determined on a 5- (from 1, comfortable, to 5, very uncomfortable) and 17- (from 0.0, unbearably cold, to 8.0, unbearably hot) point scale, respectively (Toner et al. 1986), and the rating of perceived exertion (RPE) measured using a 15-point Borg scale (from 6, very very light, to 20, very very hard) (Borg 1982). Following completion of the protocol, the participants’ towel dried and recorded NBM for the later calculation of whole-body sweat rate (WBSR, L h^−1^).

### Data analysis

Data are presented as mean ± SD (*n* = 14) unless, otherwise, indicated. All statistical analyses were carried out using the SPSS software (Version 25). All outcome variables were first checked for normality and sphericity. The Greenhouse–Geisser correction for the *F* statistic and related degrees of freedom was used when data violated sphericity. The data from the CISP were “blocked” into an average of each 10 min series of sprints/recovery sprints. A two-way repeated-measures analysis of variance (ANOVA) was performed to determine differences in dependent variables associated with performance (PP, MP; W kg^−1^) during each of the four 10 min blocks between the four mouth-rinse trials. Perceptual measures of RPE, *T*_sen_, *T*_com_, and physiological measures of *T*_rec_, *T*_skin_, and HR were compared between the four trials, and across five timepoints (0, 10, 20, 30, 40 min). WBSR and WD were analyzed using a one-way repeated-measures ANOVA between the four mouth-rinse trials. Main and interaction effects were followed up with Bonferroni adjusted post hoc comparisons. Partial Eta squared ($\eta _{{\text{p}}}^{2}$) was used as an estimate of the effect size for main and interaction effects [0.01 = small, 0.06 = medium, and 0.013 = large (Cohen 1992)]; Cohen’s *d*_av_ (*d*_av_) was used as an estimate of the effect size [0.2 = small, 0.5 = medium, and 0.8 = large (Cohen 1992)] for post hoc comparisons. The threshold for rejecting the null hypothesis was set at *P* < 0.05.

## Results

### Effect of mouth rinse on sprint performance

**Fig. 1:**
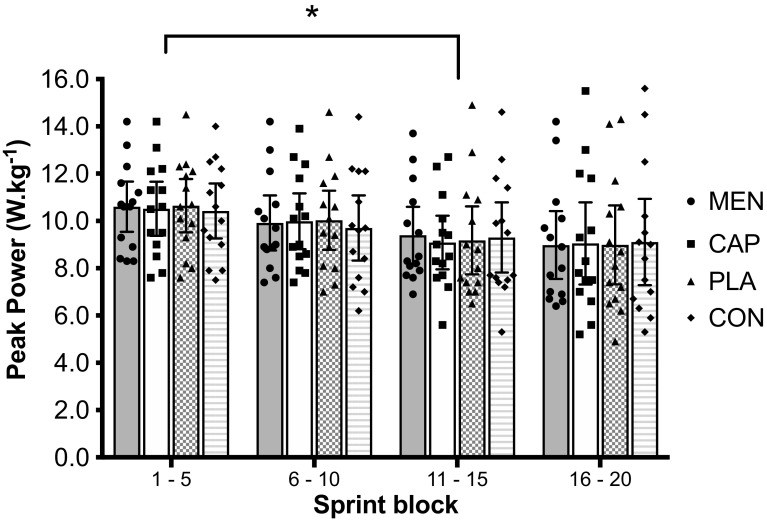
Mean ± 95% CI Peak power output during the sprint blocks across the menthol, capsaicin, carbohydrate, and water mouth-rinse conditions. Asterisk denotes difference from the previous sprint block with no difference between groups

**Fig. 2:**
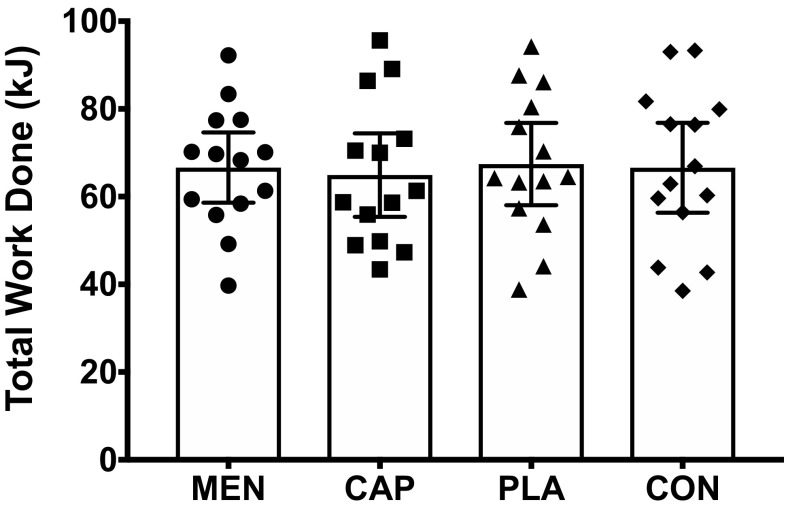
Mean ± 95% CI total work done during sprints across the menthol, capsaicin, carbohydrate, and water mouth-rinse conditions

There was no main effect of mouth rinse, or interaction effect between mouth rinse and time, on peak power (Fig. 1), mean power or total work done (Fig. 2) [PP mouth rinse: *F*_(2,27)_ < 0.1, *P* = 0.967, $\eta _{{\text{p}}}^{2}$ = 0.003, mouth rinse × time: *F*_(4,54)_ = 0.4, *P* = 0.776, $\eta _{{\text{p}}}^{2}$ = 0.034, MP mouth rinse: *F*_(3,39)_ < 0.1, *P* = 0.981, $\eta _{{\text{p}}}^{2}$ = 0.005, mouth rinse × time: *F*_(4,51)_ = 0.9, *P* = 0.447, $\eta _{{\text{p}}}^{2}$ = 0.067, work done (kJ) mouth rinse: *F*_(3,39)_ = 0.4, *P* = 0.735, $\eta _{{\text{p}}}^{2}$ = 0.032].

Peak power (W kg^−1^) and mean power (W kg^−1^) decreased during the first three blocks [main effect of time, PP: *F*_(1.3,16)_ = 10.6, *P* = 0.003, $\eta _{{\text{p}}}^{2}$ = 0.450, MP: *F*_(1,15)_ = 14.3, *P* = 0.001, $\eta _{{\text{p}}}^{2}$ = 0.524]. PP and MP were significantly lower during sprints 6–10 compared to sprints 1–5 (PP: *P* = 0.027, *d*_av_ = 0.3, MP: *P* = 0.018, *d*_av_ = 0.3), and significantly lower during sprints 11–15 compared to sprints 6–10 (PP: *P* = 0.002, *d*_av_ = 0.3, MP: *P* < 0.001, *d*_av_ = 0.3). Sprint performance data are presented in Figs. 1 and 2 with data tables included as electronic supplementary material.

### Perceptual responses to mouth rinse

There was a main effect of mouth rinse on *T*_com_ [*F*_(3,39)_ = 2.9, *P* = 0.046, $\eta _{{\text{p}}}^{2}$ = 0.183], where *T*_com_ was lower after L-menthol (2.7 ± 1.1) than after carbohydrate mouth rinse (3.2 ± 1.3, *P* = 0.042, *d*_av_ = 0.4). There was no difference between any other mouth-rinse conditions, and no interaction effect between mouth rinse and time [*F*_(6,82)_ = 0.8, *P* = 0.548, $\eta _{{\text{p}}}^{2}$ = 0.061].

**Fig. 3:**
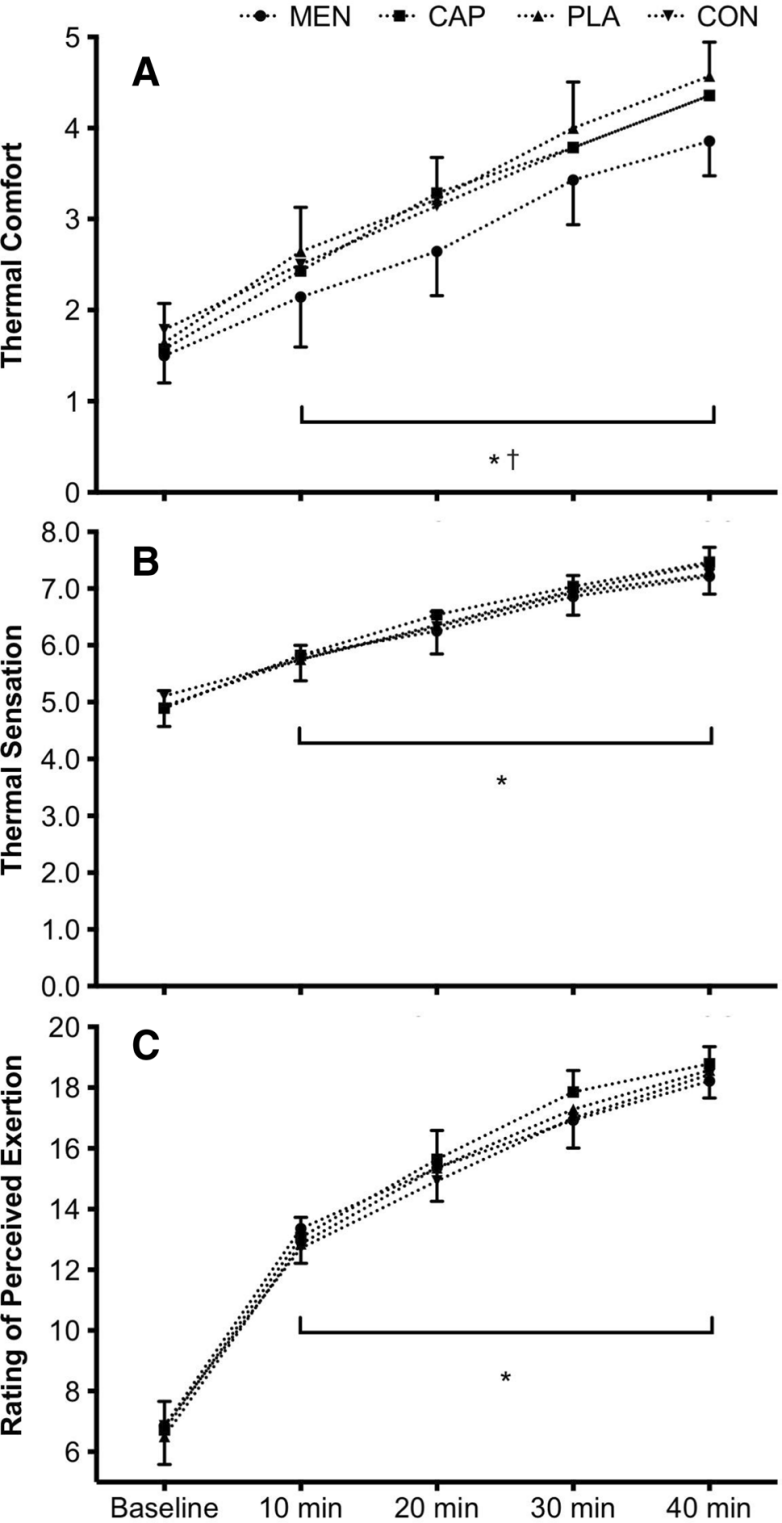
Mean ± 95% CI thermal comfort (**a**), thermal sensation (**b**), and rating of perceived exertion (**c**) across the menthol, capsaicin, carbohydrate, and water mouth-rinse conditions, for all time points. Asterisk denotes difference from the previous sprint block. Dagger denotes significant difference in menthol

*T*_com_ [*F*_(2,31)_ = 121.0, *P* < 0.0001, $\eta _{{\text{p}}}^{2}$ = 0.903], *T*_sen_ [*F*_(2,20)_ = 216.1 *P* < 0.0001, $\eta _{{\text{p}}}^{2}$ = 0.943] and RPE [*F*_(2,28)_ = 399.1, *P* < 0.0001, $\eta _{{\text{p}}}^{2}$ = 0.968] increased throughout each trial where all perceptual responses were higher than at the previous time point (*T*_com_; all *P* < 0.003, *d*_av_ > 0.6, *T*_sen_; all *P* < 0.0001, *d*_av_ > 0.7, RPE; all *P* < 0.0001, *d*_av_ > 0.7). Perceptual responses to mouth rinse are displayed in Fig. 3 with data tables included as electronic supplementary material.

There was no main effect of mouth rinse and no interaction between mouth rinse and time on *T*_sen_ or RPE [*T*_sen_ mouth rinse: *F*_(3,39)_ = 0.6, *P* = 0.639, $\eta _{{\text{p}}}^{2}$ = 0.042, mouth rinse × time: *F*_(5,66)_ = 1.2, *P* = 0.313, $\eta _{{\text{p}}}^{2}$ = 0.085, RPE mouth rinse: *F*_(2,26)_ = 0.6, *P* = 0.543, $\eta _{{\text{p}}}^{2}$ = 0.046, mouth rinse × time: *F*_(12,156)_ = 1.2, *P* = 0.289, $\eta _{{\text{p}}}^{2}$ = 0.084].

### Physiological responses to mouth rinse

**Fig. 4:**
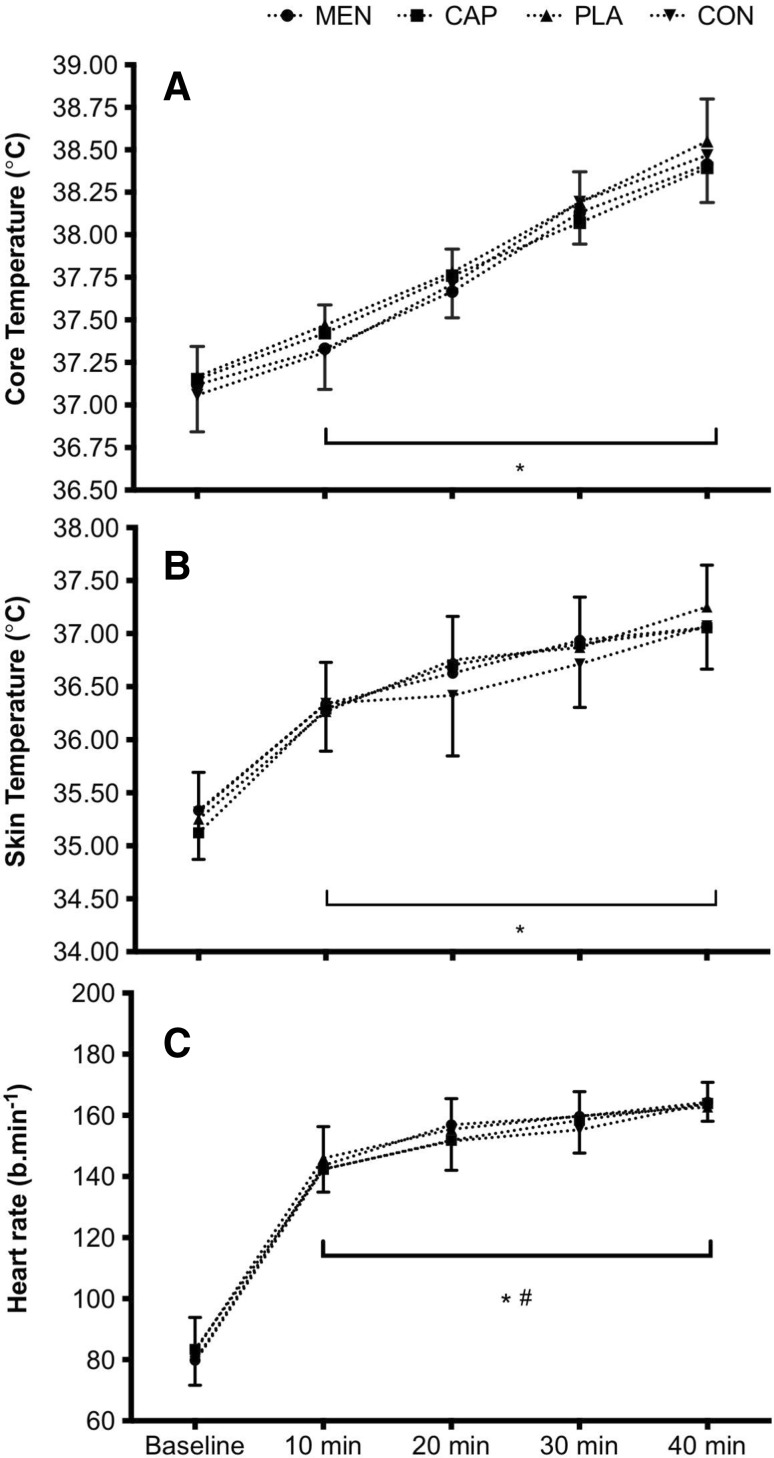
Mean ± 95% CI core temperature (**a**), skin temperature (**b**), and heart rate (**c**) across the menthol, capsaicin, carbohydrate, and water mouth-rinse conditions, for all time points. Asterisk denotes difference from the previous sprint block with no difference between groups

**Fig. 5:**
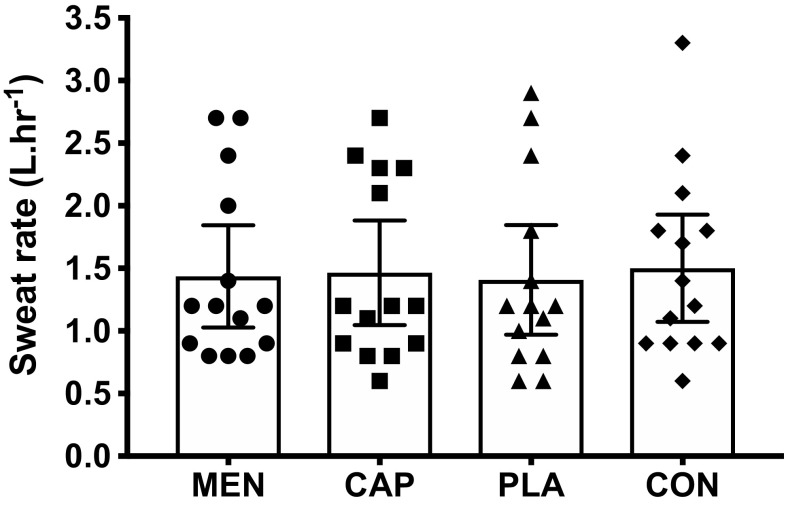
Mean ± 95% CI sweat rate across the menthol, capsaicin, carbohydrate, and water mouth-rinse conditions

There was no main effect of mouth rinse, or interaction effect between mouth rinse and time, on *T*_rec_, *T*_skin,_ HR or WBSR [*T*_rec_: mouth rinse: *F*_(3,39)_ < 0.1, *P* = 0.967, $\eta _{{\text{p}}}^{2}$ = 0.003, mouth rinse × time: *F*_(3,44)_ = 1.1, *P* = 0.355, $\eta _{{\text{p}}}^{2}$ = 0.079, *T*_skin_ mouth rinse: *F*_(3,39)_ = 0.1, *P* = 0943, $\eta _{{\text{p}}}^{2}$ = 0.010, mouth rinse × time: *F*_(3,44)_ = 0.9, *P* = 0.465, $\eta _{{\text{p}}}^{2}$ = 0.064, HR mouth rinse: *F*_(3,39)_ = 0.4, *P* = 0.769, $\eta _{{\text{p}}}^{2}$ = 0.028, mouth rinse × time: *F*_(4,56)_ = 0.5, *P* = 0.769, $\eta _{{\text{p}}}^{2}$ = 0.035, WBSR mouth rinse: *F*_(3,39)_ = 0.2, *P* = 0.925, $\eta _{{\text{p}}}^{2}$ = 0.012]. Physiological responses are presented in Figs. 4 and 5 with data tables included as electronic supplementary material.

Physiological responses are presented in Figs. 4 and 5 with data tables included as electronic supplementary material. *T*_rec_ (°C), *T*_skin,_ (°C), and HR (b min^−1^) increased over time *T*_rec_: *F*_(1,15)_ = 150.7, *P* < 0.001, $\eta _{{\text{p}}}^{2}$ = 0.921, *T*_skin_: *F*_(2,21)_ = 78.4, *P* = 0.001, $\eta _{{\text{p}}}^{2}$ = 0.858, HR: (*F*_(2,26)_ = 294.6 *P* < 0.001, $\eta _{{\text{p}}}^{2}$ = 0.958].

*T*_rec_ was higher at every time point than the previous time point (all *P* < 0.001, all *d*_av_ > 0.8). The same trend occurred for *T*_skin_ and HR (all *P* < 0.02, *d*_av_ > 0.4), though the differences between 20 min and 30 min (*T*_skin_: *P* = 0.051, *d*_av_ = 0.3, HR: *P* = 0.316, *d*_av_ = 0.3) were not statistically significant.

## Discussion

The aim of this experiment was to determine whether eliciting a cooling sensation via oral L-menthol or a warming sensation via oral capsaicin would alter intermittent sprint performance in the heat in comparison to control and placebo oral solutions. Identical physiological responses (Figs. 4, 5) between mouth-rinse conditions demonstrate equality of the physical capacity to perform intermittent sprints in the heat. Enhanced thermal comfort [− 0.5 vs PLA (*P* < 0.05), and − 0.4 vs CAP and CON] following L-menthol stimulation of the TRPM8 ion channel (Fig. 3) suggested that the participants were more ‘perceptually tolerant’ of the physiological heat stress, therefore, in line with the work of others, which had the potential to perform better given a reduction in perceived heat stress. This improved thermal perception via L-menthol did not, however, alter intermittent sprint performance (Figs. 1, 2). Opposing the experimental hypothesis, the capsaicin mouth rinse elicited no detrimental effect on performance, perception, or physiological responses with all data comparable to the placebo and control. Accordingly, the targeted TRPV1 channel was insufficiently modulated by our capsaicin rinse protocol.

Our data contrast the evidence of performance enhancement during fixed-intensity tasks following oral menthol interventions from cycling time to exhaustion/task failure experiments [+ 9% (Mündel and Jones 2010), + 8% (Flood et al. 2017) and + 6% (Jeffries et al. 2018)] and pre-loaded running time trials of 3 km and 5 km [TT; − 3% (Stevens et al. 2016) and − 3.5% (Stevens et al. 2017)]. The common theme amongst the studies demonstrating a benefit of Menthol are reports of improved thermal perception (Stevens et al. 2016, 2017; Flood et al. 2017; Jeffries et al. 2018) or reduced perceived exertion (Mündel and Jones 2010). Our data add to the equivocal findings relating to the use of oral Menthol interventions which have reported no ergogenic effects during performance trials, e.g., 400 m running TT (Sönmez et al. 2010), 20 km (Riera et al. 2014), and 30 km cycle TT (Riera et al. 2016). It is noteworthy that improved thermal perception (Riera et al. 2014) and perceived exertion (Riera et al. 2016) do not necessarily improve performance. In support of these equivocal data relating to the importance of thermal/exercise perception on performance following oral menthol, it has also been observed that menthol spray to the torso offers no ergogenic effect during 16.1 km (Barwood et al. 2015) or 40 km cycle TT performance (Barwood et al. 2012), or during a pre-loaded 5 km running TT (Barwood et al. 2014) despite improved thermal sensation. Combining menthol with neck cooling also elicits no difference in exercise performance (time to task failure) in comparison to abdominal or non-menthol neck cooling, or in comparison a control trial, in spite of improved thermal sensation [vs no intervention (Bright et al. 2018)]. The role of improved thermal or exertional perception via menthol remains equivocal with no consensus on which perceptual metric for thermal/exertion feelings is most affected by L-menthol despite their proposed importance during continuous, fixed-intensity tasks (Flood 2018). Our data highlight that, during intermittent sprint exercise, in spite of improved thermal perception of equivalent magnitudes to others [specifically thermal comfort, but not thermal sensation as per (Flood et al. 2017)], menthol elicits no ergogenic benefit.

Only one experiment has considered the role of L-menthol and subsequent manipulations in thermal perception on intermittent exercise performance (Trong et al. 2015). The intermittent exercise in this study was unaffected by L-menthol; however, the long, higher intensity intermittent bouts in this study do not reflect the activity profiles of field-based team sports making comparisons with the current protocol inappropriate. A number of other experiments have implemented supramaximal intermittent sprint exercise (similar to the CISP) and altered either thermal perception, i.e., sensation or comfort, or physiological temperature. During intermittent sprint exercise in the heat for example, ice slurry consumption reduced core temperature (start of protocol − 0.5 °C, end of protocol − 0.3 °C) and thermal sensation (− 3), but did not influence the total distance covered or speed during jog, run, and sprint phases of the protocol (Gerrett et al. 2017). As skin temperature and RPE were not different, the authors concluded, in congruence with the previous work (Duffield and Marino 2007), that combined alterations in core and skin temperature are necessary for influencing intermittent sprint performance in the heat (Gerrett et al. 2017). The CISP has been utilized in experiments where various manipulations of temperature perception and actual temperature have occurred. It has been observed that, in comparison to control conditions, a cooling intervention using ice packs (local cooling of exercising muscle) can reduce muscle temperature (ice packs − 0.6 °C vs control), core temperature (− 0.2 °C), and heart rate (− 7 b min^−1^ vs control) at the end of a CISP performed in hot conditions (Castle et al. 2006). The result of these temperature manipulations was increased PP (+ 4%) and WD (+ 2%) in the ice packs only condition (Castle et al. 2006). These performance data, coupled with no differences in either RPE or *T*_sens_ between cooling and control trials, suggest that the performance ability during intermittent sprints in the heat is more closely related to physiological responses, and particularly local muscle temperature, rather than perceived temperature. This provides an explanation for why, despite improved thermal comfort with L-menthol, intermittent sprint performance was unchanged in the present study. Support for this mechanism can be found when examining the independent impact of heat acclimation, and pre-cooling interventions on CISP in the heat. Only when implementing a heat acclimation intervention that induces reductions in core temperature (− 0.4 °C), heart rate (− 18 b min^−1^) and thermal sensation (− 1.0) did PP improve in the heat (+ 2%) with pre-cooling (thermal and non-thermal cooling) offering no additional benefit (Castle et al. 2011). This appears a different mechanism to that observed during 5000 m running in the heat where pre-cooling demonstrates an ergogenic effect independently (TT duration − 3.7%), and when used in conjunction with heat acclimation (TT duration − 7.0%) greater performance enhancement than heat acclimation alone (TT duration − 6.6%) (James et al. 2018). These findings highlight that, whilst physiological intervention is a priority for intermittent sprinting in the heat (Castle et al. 2006, 2011), physiological and perceptual manipulations should be conferred for continuous intensity, endurance performance in the heat (James et al. 2017), suggesting that there may still be benefits associated with oral menthol in this domain (Stevens and Best 2017; Flood 2018).

### Experimental considerations and future directions

In light of the small adjustments to thermal comfort, and no observed change in thermal sensation, it could be suggested that despite replicating dosages utilized in the previous experimental work (Mündel and Jones 2010; Stevens et al. 2016), the concentration of L-menthol (0.01%) was insufficient to elicit changes in behavioral thermoregulation. This comment can also be made in regard to the exploratory inclusion of our capsaicin rinse. Recent experimental work has subsequently identified that the concentration of L-menthol (concentrations range 0.05–0.105% L-menthol at 0.05% increments when dissolved in ethanol, rather than water) being mouth rinsed does not alter thermal perception within individuals (Best et al. 2018) . Accordingly, although the rinse frequency in the present study replicates the timing used by Mündel and Jones (2010), a more frequent rinse in line with Stevens et al. (2016) may be ergogenic during intermittent sprint and warrants further investigation. Understanding the impact of alterations in L-menthol mouth-rinse temperature may be required to understand the role this plays in intermittent sprint performance given that our experiment utilized a drink temperature similar to deep body temperature to minimize the influence of visceral temperature modulation (Morris et al. 2018), but (for experimental control) this temperature was greater than that likely consumed in actual competition and this may be one reason for different findings compared to studies using cooler mouth rinses (Stevens and Best 2017). It is likely that the capsaicin ‘dose’ used in this experiment was suboptimal to achieve our experimental aim. The use of capsaicin mouth rinse (to make mechanistic inference rather than as an ergogenic aid) to increase thermal perception, therefore, requires further refinement allied to application, concentration, and frequency given the lack of a response across dependent variables, an opposing finding to that of topical capsaicin (Schlader et al. 2011). The present experiment did not utilize sufficient techniques to understand the mechanisms, and level at which L-menthol cooling is ergogenic. To elucidate this, techniques such as peripheral nerve stimulation and transcranial magnetic stimulation (Goodall et al. 2014; Twomey et al. 2017) may be used to determine central and peripheral components to the reduction in workload associated with intermittent sprint exercise in the heat, with and without alterations in perception via L-menthol. The intensity of continuous endurance activity is closer to the submaximal intensity proposed as subject to influence via pre-cooling (Duffield and Marino 2007), than the supramaximal sprint efforts within the CISP suggesting that perceived temperature plays a lesser role in modulated short sprint exercise of a fixed number and frequency. Performance during submaximal phases of an intermittent sprint protocol is most likely to be influenced by cooling, rather than the sprint itself (Duffield and Marino 2007), Regrettably, this was not quantifiable in the present experiment given that the fixed-intensity nature of our active recovery protocol but the notion that peak sprint performance is less likely to be influenced by actual/perceptual cooling was observed (Fig. 1). These comments raise an important point relating to the protocol used in the present study. Whilst the CISP is a reliable and valid protocol to determine the physiological responses to intermittent sprinting (Hayes et al. 2012, 2014), the specific task structure is such that the CISP is closed in nature. This closed task potentially creates an experimental artifact, whereby participants are not able to sprint freely (in frequency or duration). Future work should consider the benefits of an ‘open-ended’ task, e.g., devising a protocol with an undefined sprint duration, or participant regulated sprint frequency, performed on a non-motorized treadmill where participants could pace independently in response to non-thermal cooling interventions in the heat. This approach is such that it may facilitate different responses due to the elevation in pacing associated with these tasks, differentiating it from the CISP which implements fixed duration and fixed frequency of sprinting. In light of alterations in both low- and high-intensity running with heat stress (Konefal et al. 2014; Nassis et al. 2015; Watanabe et al. 2017), changes in these movement velocities could also be quantified using this approach in a similar manner to other work (Gerrett et al. 2017). Finally, the CISP is only 40 min in duration and, therefore, only replicates the first half of a field-based team sport. Future work should, therefore, extend the task duration to more closely replicate the team sport of interest (Turner et al. 2014), in elite team sport players.

### Practical implications

These data suggest that the ergogenic potential of L-menthol associated with endurance tasks does not extend to intermittent sprint performance (of 40 min) in very hot-temperature conditions (~ 40 °C). This is in spite of the magnitude in alteration in thermal perception in the present experiment being congruous with others (Stevens et al. 2017; Flood et al. 2017), meaning that our null finding relating to performance was not simply a result of a null finding relating to altering thermal perception as is the case with the capsaicin data. As such, rather than seeking perceptual manipulations alone, individuals seeking to use an intervention to enhance intermittent sprint performance, e.g., team sport players, should preference either pre-cooling, or mid (per) cooling interventions as an acute ergogenic aid (Castle et al. 2006; Luomala et al. 2012; Sunderland et al. 2015), or a heat acclimation strategy, e.g., the preferable isothermic approach (Racinais et al. 2015a; Pryor et al. 2018), to induce enhanced physiological responses to intermittent sprinting as part of a chronic intervention (Sunderland et al. 2008; Castle et al. 2011) in a manner that minimizes training disruption (Gibson et al. 2015).

## Conclusion

Mouth rinsing with L-menthol improves thermal comfort during 40 min of intermittent sprint exercise in the heat, but this does not appear to alter ISP. Capsaicin did not alter thermal perception or ISP. No mouth rinse utilized changed the physiological responses to ISP in the heat. Alterations to the intervention, e.g., mouth-rinse frequency/duration subsequently leading to a greater change in thermal perception, or an open loop ISP may induce ergogenic responses, however, based on the present data; the reduction in ISP over time in hot conditions is not influenced by altering thermal perception.

## Electronic supplementary material

Below is the link to the electronic supplementary material.

Supplementary material 1 (DOCX 26 KB)

## Abbreviations

*T*_rec_: Core temperature
CISP: Cycling intermittent sprint protocol
HR: Heart rate
ISP: Intermittent sprint performance
*W*_max_: Maximum aerobic power output
MP: Mean power
NBM: Nude-body mass
$\dot {V}{{\text{O}}_{2{\text{peak}}}}$: Peak oxygen uptake
PP: Peak power
RPE: Rating of perceived exertion
*T*_skin_: Skin temperature
*T*_com_: Thermal comfort
*T*_sens_: Thermal sensation
TT: Time trial
TRP: Transient receptor potential channels
TRPM8: TRP ion-channel melastatin 8
TRPV1: TRP ion-channel vanilloid 1
WBSR: Whole-body sweat rate
WD: Work done
