# Supplementary material for: Intermittent sprint performance in the heat is not altered by augmenting thermal perception via L-menthol or capsaicin mouth rinses
Source: Eur J Appl Physiol. 2018 Dec 22;119(3):653–64. doi: 10.1007/s00421-018-4055-0 (PMC6394657; doi:10.1007/s00421-018-4055-0)
Supplement: Supplementary file 1 — Supplementary material 1 (DOCX 26 KB) [file 421_2018_4055_MOESM1_ESM.docx]

| Supplementary Table S1. Mean±SD Sprint performance during blocked sprints | | | | | |
| --- | --- | --- | --- | --- | --- |
|  | Rinse | Sprints | | | |
|  |  | 1-5 | 6-10* | 11-15* | 16-20* |
| Peak Power (W.kg^-1^) | Menthol | 10.6±1.8 | 9.9±2.0 | 9.4±2.1 | 9.0±2.5 |
|  | Capsaicin | 10.5±2.0 | 10.0±2.1 | 9.1±2.0 | 9.0±3.0 |
|  | Carbohydrate | 10.6±2.0 | 10.0±2.2 | 9.2±2.5 | 9.0±2.9 |
|  | Water | 10.4±2.0 | 9.7±2.4 | 9.3±2.6 | 9.1±3.2 |
| Mean Power  (W.kg^-1^) | Menthol | 8.7±1.4 | 8.0±1.8 | 7.5±1.7 | 6.9±2.2 |
|  | Capsaicin | 8.7±1.9 | 8.0±2.1 | 7.1±2.2 | 7.2±2.8 |
|  | Carbohydrate | 8.7±1.8 | 8.3±2.0 | 7.5±2.1 | 7.1±2.6 |
|  | Water | 8.5±1.8 | 8.0±2.2 | 7.5±2.5 | 7.2±2.8 |
| Total work done  (kJ) | Menthol | 66.6±13.9 | | | |
|  | Capsaicin | 64.9±16.5 | | | |
|  | Carbohydrate | 67.4±16.2 | | | |
|  | Water | 66.6±17.7 | | | |
| Note total work done is the mean of all sprints. * Denotes difference from previous sprint block with no difference between groups | | | | | |

| Supplementary Table S2. Mean+SD perceptual measures across all time points | | | | | | |
| --- | --- | --- | --- | --- | --- | --- |
|  | Rinse | Time (minutes) | | | | |
|  |  | Start | 10 | 20 | 30 | 40 |
| Thermal Comfort | Menthol | 1.5±0.5 | 2.1±0.9 | 2.6±0.8 | 3.4±0.9 | 3.9±0.7 |
|  | Capsaicin | 1.6±0.8 | 2.4±0.8 | 3.3±0.9 | 3.8±0.8 | 4.4±0.6 |
|  | Carbohydrate | 1.6±0.7 | 2.6±0.8 | 3.2±0.8 | 4.0±0.9 | 4.6±0.6 |
|  | Water | 1.8±0.8 | 2.5±1.0 | 3.1±1.0 | 3.8±1.1 | 4.4±1.0 |
| Thermal Sensation | Menthol | 4.9±0.6 | 5.8±0.8 | 6.3±0.7 | 6.9±0.6 | 7.2±0.5 |
|  | Capsaicin | 4.9±0.4 | 5.8±0.3 | 6.5±0.5 | 7.0±0.4 | 7.5±0.4 |
|  | Carbohydrate | 4.9±0.5 | 5.8±0.4 | 6.4±0.4 | 7.0±0.5 | 7.4±0.5 |
|  | Water | 5.1±0.4 | 5.8±0.6 | 6.3±0.7 | 6.9±0.7 | 7.3±0.7 |
| RPE | Menthol | 7±2 | 13±2 | 15±2 | 17±2 | 18±1 |
|  | Capsaicin | 7±2 | 13±1 | 16±2 | 18±1 | 19±1 |
|  | Carbohydrate | 7±1 | 13±1 | 15±2 | 17±1 | 19±1 |
|  | Water | 7±2 | 13±1 | 15±1 | 17±1 | 18±1 |
|  | | | | | | |

| Supplementary Table S3. Mean±SD physiological measures for all time points | | | | | | |
| --- | --- | --- | --- | --- | --- | --- |
|  | Rinse | Time (minutes) | | | | |
|  |  | Start | 10 * | 20 * | 30 * | 40 * |
| T_rec_ (°C) | Menthol | 37.12±0.39 | 37.33±0.32 | 37.67±0.38 | 38.13±0.34 | 38.41±0.36 |
|  | Capsaicin | 37.15±0.29 | 37.42±0.24 | 37.76±0.34 | 38.07±0.45 | 38.39±0.49 |
|  | Carbohydrate | 37.17±0.31 | 37.47±0.21 | 37.78±0.24 | 38.19±0.31 | 38.55±0.43 |
|  | Water | 37.06±0.38 | 37.31±0.38 | 37.71±0.34 | 38.19±0.43 | 38.47±0.48 |
| T_skin_ (°C) | Menthol | 35.33±0.54 | 36.34±0.59 | 36.62±0.48 | 36.93±0.57 | 37.05±0.75 |
|  | Capsaicin | 35.12±1.14 | 36.29±0.78 | 36.70±0.88 | 36.90±0.70 | 37.05±0.59 |
|  | Carbohydrate | 35.25±0.76 | 36.26±0.80 | 36.75±0.71 | 36.87±0.82 | 37.25±0.68 |
|  | Water | 35.22±0.77 | 36.34±0.78 | 36.42±0.99 | 36.71±0.71 | 37.07±0.71 |
| Heart rate  (b.min^-1^) | Menthol | 80±13 | 144±21 | 157±19 | 160±15 | 164±16 |
|  | Capsaicin | 83±22 | 142±15 | 152±16 | 158±13 | 164±12 |
|  | Carbohydrate | 83±19 | 146±18 | 155±17 | 160±14 | 163±14 |
|  | Water | 79±13 | 142±13 | 152±17 | 155±13 | 1640±10 |
| Whole Body Sweat Rate  (L.hr^-1^) | Menthol | 1.4±0.7* | | | | |
|  | Capsaicin | 1.4±0.7* | | | | |
|  | Carbohydrate | 1.40±0.8* | | | | |
|  | Water | 1.5±0.7* | | | | |
| Note WBSR is the mean across all time points. * Denotes difference from previous sprint block with no difference between groups | | | | | | |
